# Cryo-electron microscopy structures of pyrene-labeled ADP-P_i_- and ADP-actin filaments

**DOI:** 10.1038/s41467-020-19762-1

**Published:** 2020-11-19

**Authors:** Steven Z. Chou, Thomas D. Pollard

**Affiliations:** 1 Department of Molecular Cellular and Developmental Biology, Yale University, PO Box 208103, New Haven, CT 06520-8103 USA; 2 Department of Molecular Biophysics and Biochemistry, Yale University, PO Box 208103, New Haven, CT 06520-8103 USA; 3 Department of Cell Biology, Yale University, PO Box 208103, New Haven, CT 06520-8103 USA

**Keywords:** Chemical modification, Cryoelectron microscopy, Supramolecular assembly

## Abstract

Since the fluorescent reagent N-(1-pyrene)iodoacetamide was first used to label skeletal muscle actin in 1981, the pyrene-labeled actin has become the most widely employed tool to measure the kinetics of actin polymerization and the interaction between actin and actin-binding proteins. Here we report high-resolution cryo-electron microscopy structures of actin filaments with N-1-pyrene conjugated to cysteine 374 and either ADP (3.2 Å) or ADP-phosphate (3.0 Å) in the active site. Polymerization buries pyrene in a hydrophobic cavity between subunits along the long-pitch helix with only minor differences in conformation compared with native actin filaments. These structures explain how polymerization increases the fluorescence 20-fold, how myosin and cofilin binding to filaments reduces the fluorescence, and how profilin binding to actin monomers increases the fluorescence.

## Abstract (web summary)

For almost forty years, N-(1-pyrene) iodoacetamide has been used to label actin at C374, but the mechanisms of the fluorescence changes are still unknown due to the lack of structural information. Here authors provide cryo-EM structures of actin filaments with N-1-pyrene conjugated to cysteine 374 and either ADP or ADP-phosphate in the active site.

## Introduction

Actin, one of the most abundant proteins in all eukaryotic cells, can polymerize into long filaments, one of the three major types of filaments that make up the cytoskeleton.

These filament structures give the cell its shape and help organize the parts of the cell. The maintenance or change of local and/or global cell structures is important to most cellular processes. At the molecular level, many of these cellular processes are achieved by the polymerization and depolymerization of actin filaments and the interaction between actin and actin-binding proteins. To study these molecular events, many fluorescent reagents have been developed for labeling actin either covalently or non-covalently.

For almost forty years, N-(1-pyrene) iodoacetamide has been used to covalently label actin at C374, the only solvent-accessible of five cysteine residues^1^. Thousands of studies have used the fluorescence of pyrenyl-actin to measure actin polymerization^1,2^ and binding of myosin^1^, profilin^3^, and cofilin^4,5^ to actin filaments and monomers. The mechanisms of the fluorescence changes are still unknown due to the lack of structural information.

Here we provide high-resolution cryo-electron microscopy structures of pyrene-labeled ADP-P_i_- and ADP-actin filaments, which not only explain these mechanisms but also show how the presence of pyrene modifies the filament structure in small ways and why other dyes coupled to C374 compromise polymerization. We also report that phosphate-binding increases the fluorescence of Mg-ADP-pyrenyl-actin filaments.

## Results

### Cryo-EM structures of pyrenyl-actin filaments

**Fig. 1:**
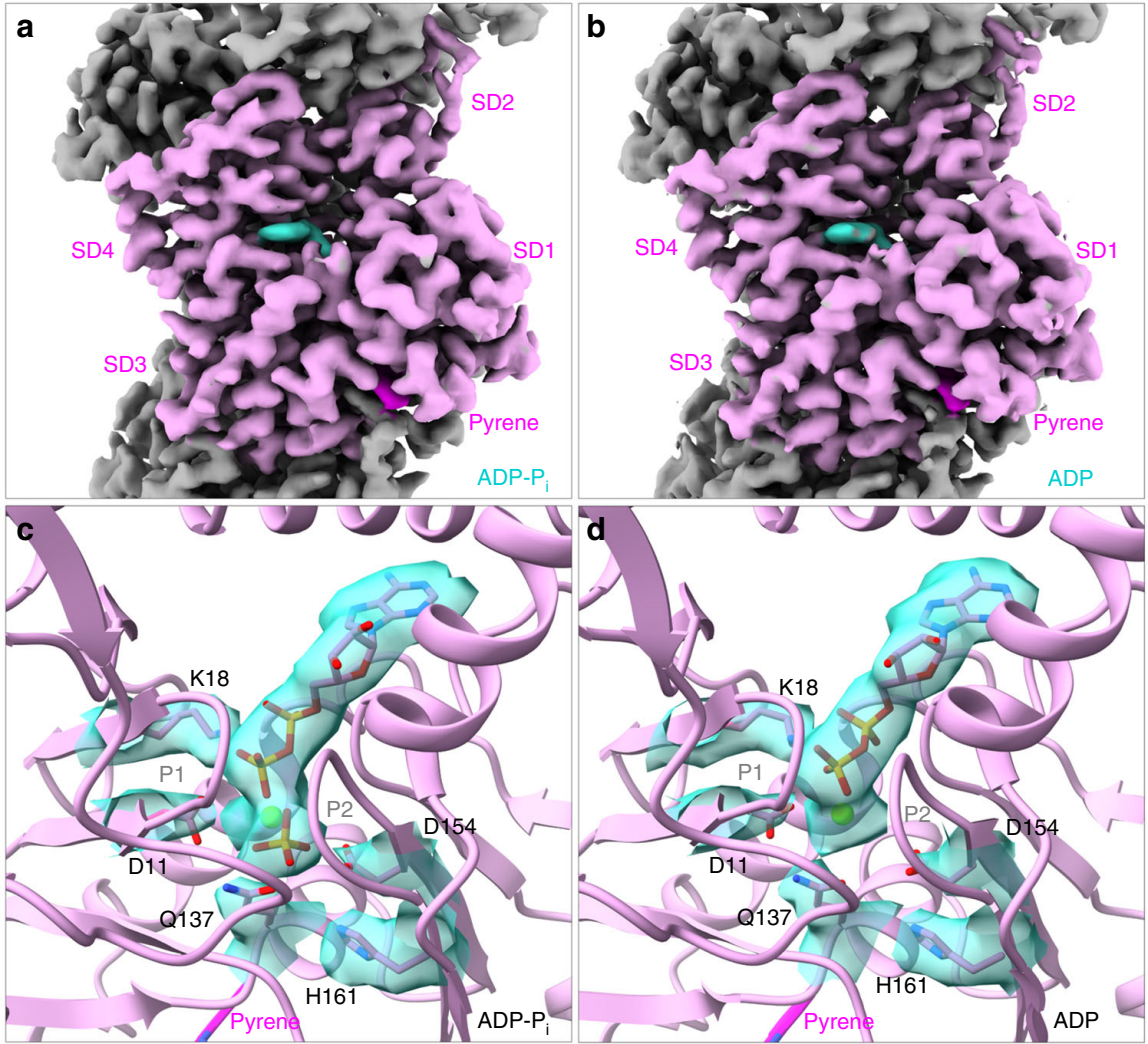
Electron potential maps and ribbon diagrams of pyrenyl-actin filaments with (a, c) bound Mg-ADP-P_i_ or (b, d) bound Mg-ADP. **a**, **b** Maps with highlighted densities of the nucleotide (turquoise) and pyrene (magenta) in one subunit (plum). Neighboring subunits are gray. The barbed end is at the bottom. The four subdomains are labeled as SD1-4. **c**, **d** Ribbon models with the densities and stick figures of the bound nucleotides and a few key neighboring side chains. P1 (residues 11–16) and P2 (residues 154–161) are the two phosphate-binding loops.

We collected 1560 movies of Mg-ADP-P_i_-pyrenyl-actin filaments and 1980 movies of Mg-ADP-pyrenyl-actin filaments on single grids in a consecutive session. After drift correction, each movie was summed into an electron micrograph before further processing (Supplementary Fig. 1). Image processing of electron micrographs of 411,301 particles of Mg-ADP-P_i_-pyrenyl-actin filaments produced an electron potential map with an overall resolution of 3.0 Å and 240,254 particles of Mg-ADP-pyrenyl-actin filaments gave a map with an overall resolution of 3.2 Å (Supplementary Fig. 2; Supplementary Table 1). The overall resolutions estimated using Fourier shell correlation (FSC) and layer-line images agreed well with map local resolutions (Supplementary Figs. 2 and 3). These maps showed the positions of most side chains, the conjugated pyrene, bound nucleotides, and associated Mg^2+^ and allowed unambiguous model building (Fig. 1). The helical parameters (rise and twist) of pyrenyl-actin filaments are nearly identical to native actin filaments in both the ADP-P_i_ and ADP states (Supplementary Table 2). The conformations of the bound nucleotides and their surrounding residues are the same in pyrenyl-actin filaments and native actin filaments (Fig. 1c, d).

**Fig. 2:**
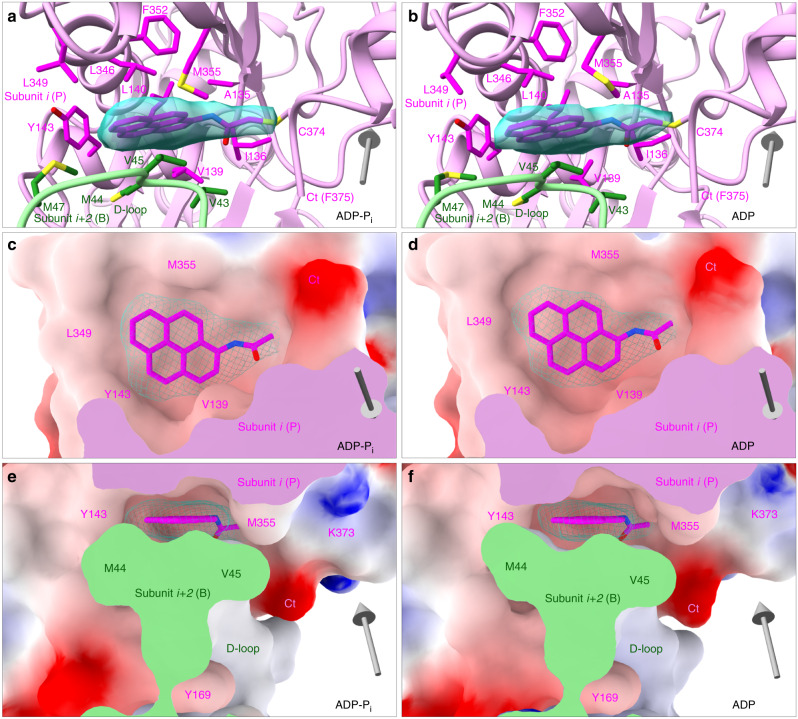
N-(1-pyrene) conjugated to C374 in a hydrophobic pocket between two subunits along the long-pitch helix of the actin filament. EM map densities of N-(1-pyrene) are shown as turquoise mesh surfaces and N-(1-pyrene) as magenta stick figures. Gray arrows show the filament orientation with arrowheads pointing to the pointed end. **a**, **c**, **e** Filaments with bound Mg-ADP-P_i_; **b**, **d**, **f** filaments with bound Mg-ADP. **a**, **b** Backbone ribbon diagrams. The actin subunit toward the pointed end is colored plum and the subunit toward the barbed end is light green. **c**, **d**, **e**, **f** Electrostatic potential surfaces (blue: positive; red: negative) of interacting actin subunits. **c**, **d** Face views; **e**, **f** side views. Neighboring subunits in (**c**) and (**d**) are omitted for clarity. The cut surfaces of the two actin subunits in the section views are colored in plum and light green.

The maps of both ADP-P_i_- and ADP-pyrenyl-actin filaments show a clear, continuous extra density for the conjugated pyrene group (Fig. 2a, b). The pyrene group conjugated to C374 is largely sandwiched in a fixed conformation between two adjacent actin subunits along the long-pitch helix between the barbed end groove of the subunit near the pointed end (called the P subunit hereafter) and D-loop of the subunit near the barbed end (called the B subunit hereafter). Large unidentified densities also appeared in this location in maps of actin filaments with bound ADP-BeF_x_ and bound jasplakinolide with either ADP-P_i_ or ADP published by Merino et al.^6^

The pyrene group in the filaments sits snugly in a hydrophobic pocket (Fig. 2c–f). Electrostatic potential maps calculated from the refined models show that the pyrene group is surrounded by the hydrophobic residues A135, I136, V139, L140, L346, L349, F352, and M355 in the barbed end groove of the P subunit and V43, M44, V45, M47 of the D-loop of the B subunit. This environment differs from the actin monomer where the pyrene group of C374 is exposed, at least in part, to the water solvent. This change of environment likely explains why the fluorescence intensity of pyrenyl actin increases ~20-fold upon polymerization.

### Structural difference between pyrenyl and native actin filaments

**Fig. 3:**
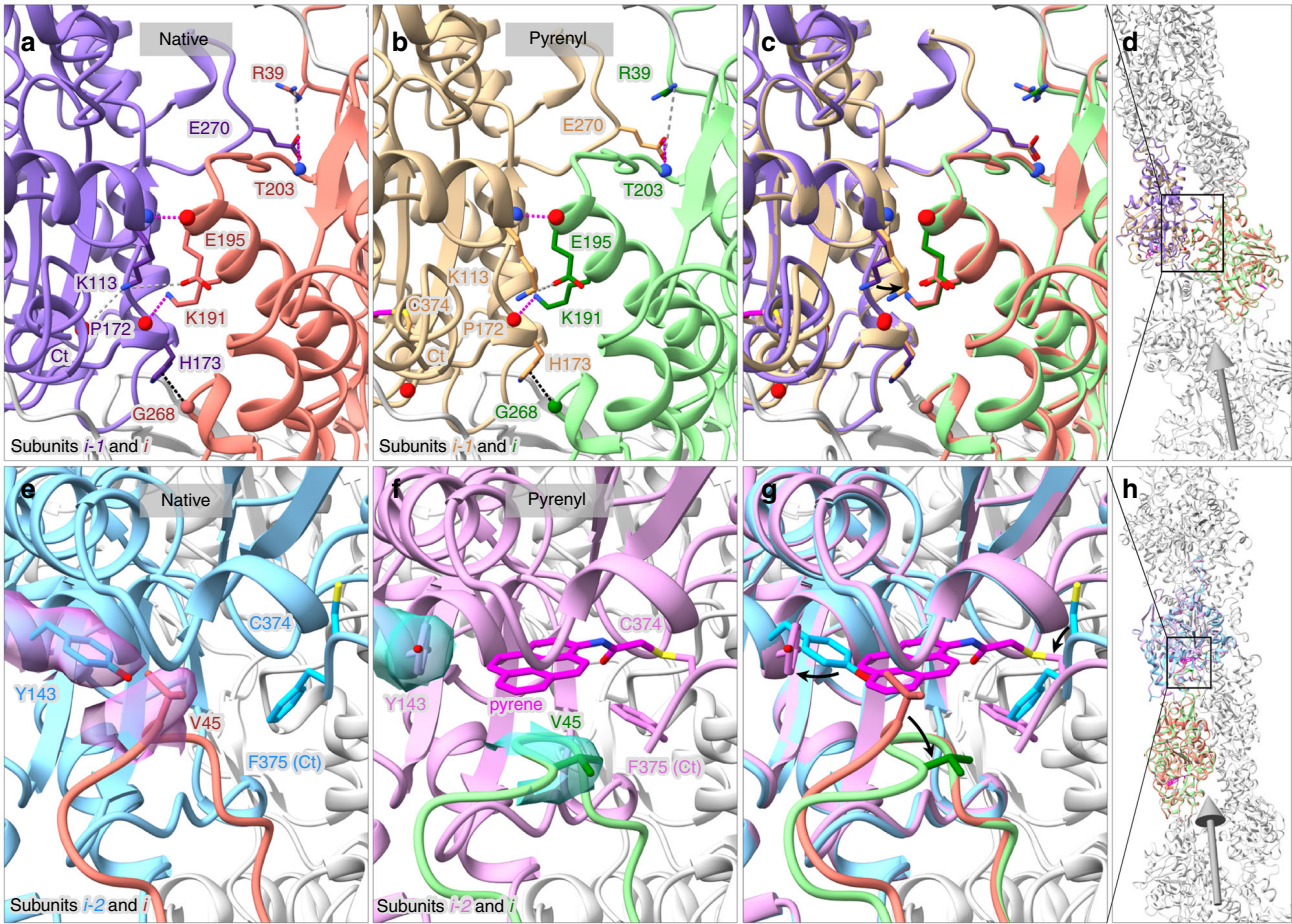
Influence of pyrene labeling of C374 on subunit interactions in actin filaments. Ribbon diagrams have stick figures of important side chains and balls for important backbone nitrogen atoms (blue), oxygen atoms (red), and C_α_ atoms (green or salmon). Gray arrows on the right column have their arrowheads pointing along the filament axis toward the pointed end. Subunits in native actin filaments are colored sky blue (subunit *i-2*), medium purple (subunit *i-1*), and salmon (subunit *i*); subunits in pyrenyl-actin filaments are colored plum (subunit *i-2*), tan (subunit *i-1*), and light green (subunit *i*). Dashed lines show charge-charge interactions (gray), hydrogen bonds (magenta), and *van der Waals* contacts (black). **a**–**d** Most lateral interactions along the short-pitch helix are the same without and with pyrene, but in pyrenyl-actin the side chain of K113 rotates (black arrow) and loses an electrostatic interaction with its own C-terminal carboxyl group while retaining an electrostatic interaction with the side chain of E195 of the neighboring subunit. **e**–**h** Effects of pyrene labeling on interactions along the long-pitch helix. Black arrows mark how pyrene causes localized changes in the conformations of V45 in the D-loop and the side chain of Y143 in the adjacent subunit. Insertion of pyrene between two actin subunits displaces the C_α_ atom of V45 towards the filament barbed end by 2.9 Å and rotation of the side chain 44.1° without disturbing other interactions between D-loop and the neighboring subunit. Pyrene twists the aromatic ring of Y143 about 72.3°. Structures of native actin filaments are from our previous study^7^, and those of pyrenyl-actin filaments are from this study.

Superimposition of the maps and models of the pyrenyl-actin filaments from this study and native actin filaments from our previous study^7^ showed that the presence of the pyrene group has a larger impact on longitudinal than lateral interactions between the subunits (Fig. 3). Pyrene displaces by small amounts the side chains of Y143 and V45 and the backbones of residues 44–48 of the D-loop of the B subunit, leaving residues 40–43 and 49–50 at either end of the D-loop in place (Fig. 3g). The pyrene group attached to the side chain of C374 also changes the local conformations of the C-terminal residues 373–375. The only difference in lateral interactions between subunits in the presence of pyrene was the movement of the side chain of K113 from about 5.0 Å to its own C-terminal carboxyl group to about 11.0 Å.

### Effect of phosphate in the nucleotide-binding site of ADP-actin filaments on labeling with N-(1-pyrene) iodoacetamide and the fluorescence intensity of pyrenyl-actin filaments

**Fig. 4:**
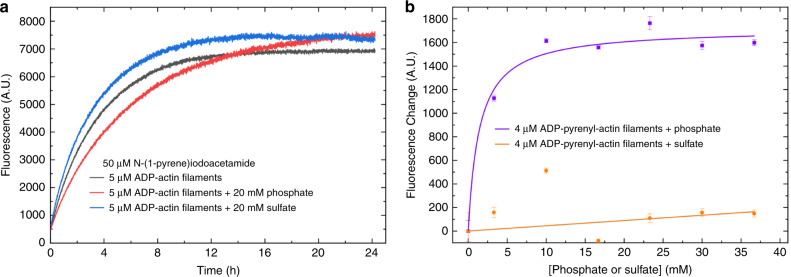
Interactions between phosphate in the nucleotide-binding site and pyrene. **a** Phosphate slows the labeling of ADP-actin filaments with N-(1-pyrene)iodoacetamide. Solutions of Mg-ADP-actin filaments were polymerized from 5 µM monomers overnight at 4 °C in 100 mM KCl; 1 mM MgCl_2_; 10 mM imidazole, pH 7.0; 0.3 mM ADP; 3 mM NaN_3_ and preincubated at room temperature with the same volume of either water, 20 mM potassium phosphate or 20 mM potassium sulfate before adding 50 µM N-(1-pyrene)iodoacetamide. The basal fluorescence is from the free N-(1-pyrene)iodoacetamide, and the fluorescence increase is due to the conjugation of N-(1-pyrene)iodoacetamide to the sidechain of C374 in actin filaments. **b** Effect of phosphate in the buffer on the fluorescence of Mg-ADP-pyrenyl-actin filaments. The fluorescence change in each data point was calculated by subtracting the fluorescence of 120 µL of Mg-ADP-pyrenyl-actin filaments (polymerized from 5 µM monomers) preincubated with 30 µL of water from the fluorescence of 120 µL of Mg-ADP-pyrenyl-actin filaments (polymerized from 5 µM monomers) preincubated with the same volume of phosphate or sulfate. Each sample was incubated for ~1 hour before the measurements. Error bars indicate the standard deviations of five readings on the same sample. The Y-axes are in arbitrary units (A.U.). Data are presented as mean values +/− SD. Source data are provided as a Source Data file.

The fluorescence emission of N-(1-pyrene) iodoacetamide is higher after conjugating to actin filaments (Fig. 4a). The conjugation reaction is very slow with half times >2 h, indicating that C374 is poorly accessible. The solvent-accessible surface area (SASA) of the side chain of C374 is ~0.1 Å^2^ in native ADP-P_i_-actin filaments, ~6.2 Å^2^ in native ADP-actin filaments^7^ and ~99.5 Å^2^ for a free cysteine. The low SASA of C374 in both ADP-P_i_- and ADP-actin filaments, explains why conjugation with N-(1-pyrene)iodoacetamide takes hours rather than minutes and why phosphate slows the reaction by a small amount. The reaction rates are consistent with the sizes of SASA in direction, but not simply in proportion.

**Fig. 5:**
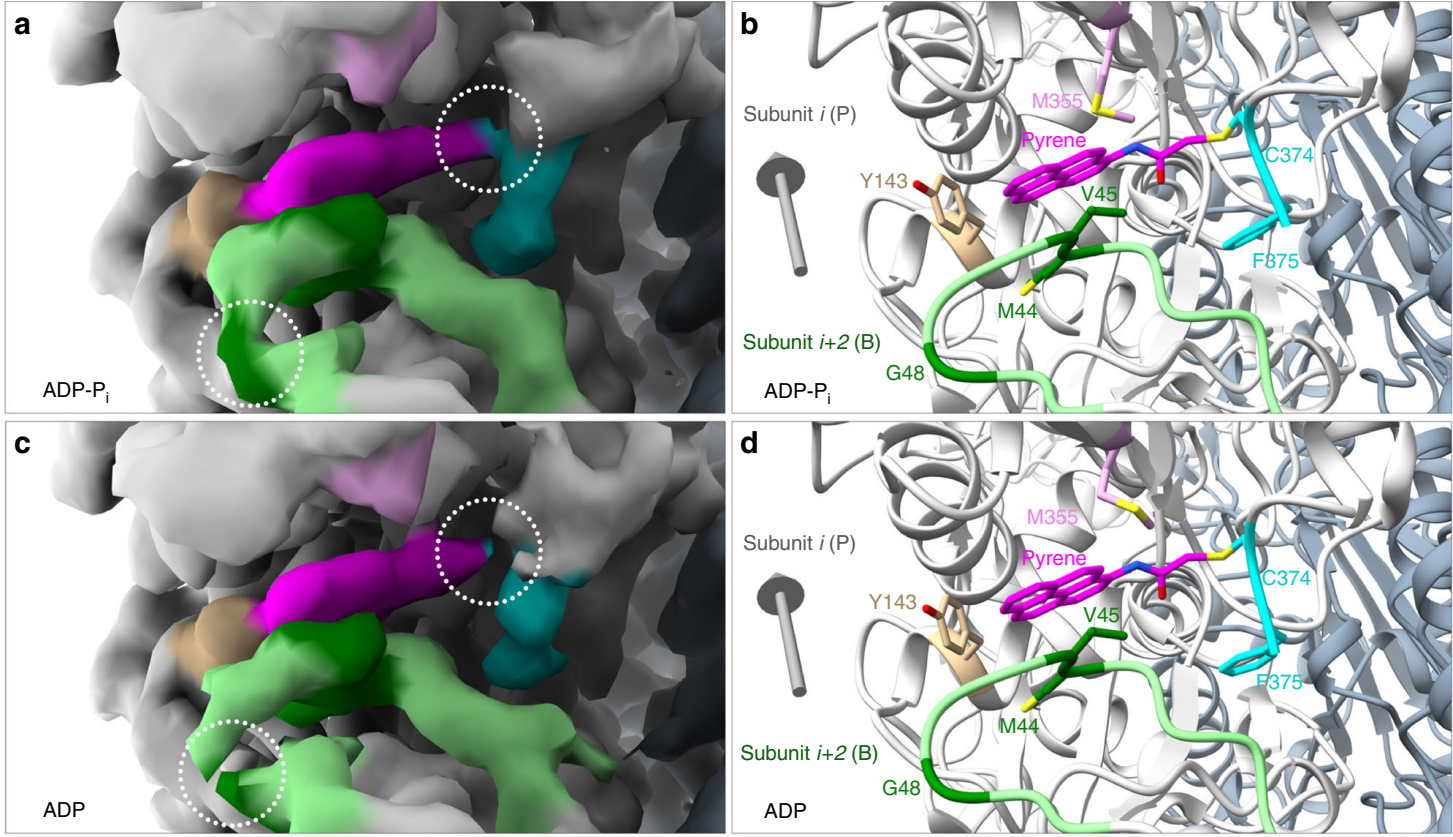
Subtle differences in the pyrene and surrounding residues in (a, b) Mg-ADP-P_i_- and (c, d) Mg-ADP-pyrenyl-actin filaments with map densities on the left and ribbon diagrams on the right. Both maps are contoured at the same level (0.015 V). Color code: actin subunits, light gray in one strand and slate gray in the other strand; D-loop (residues 40–50) of the adjacent actin subunit, light green with residues M44, V45, and G48, dark green; pyrene, magenta; C-terminus including C374 and F375, cyan; Y143, tan; M355, plum. Gray arrows are orientated with their heads pointing to the pointed end of the filaments. In the ADP-actin filament (**c**), note the lower/broken density for G48 and the connection between pyrene and the side chain of C374.

The fluorescence intensity of Mg-ADP-pyrenyl-actin filaments increases with the concentration of phosphate in the buffer (Fig. 4b) corresponding to a K_d_ < 10 mM, similar to other measurements of the affinity of the filaments for phosphate^8^. We searched for factors that might explain the effect of phosphate on the fluorescence of Mg-ADP-pyrenyl-actin filaments. The structures are very similar; RMSDs between the alpha-carbons of the models for Mg-ADP-P_i_- and Mg-ADP-pyrenyl-actin filaments were only 0.35 Å, smaller than the RMSDs of native actin filaments with the two nucleotides. However, we found two differences that might contribute to the fluorescence difference. The map densities for the connection between the side chain of C374 and pyrene, F375, and the backbone of G48 were weaker in Mg-ADP-pyrenyl-actin filaments than Mg-ADP-P_i_-pyrenyl-actin filaments (Fig. 5a, c). Furthermore, the B-factors, a rough measure of the isotropic displacements of atoms, of K373, C374, and F375 were ~7.3% higher for subunits in ADP-pyrenyl-actin filament than in ADP-P_i_-pyrenyl-actin filament, while the B-factors of the D-loop residues similar (Supplementary Fig. 4). These small differences indicate that the longitudinal interface between the B and P subunits may be less well ordered in Mg-ADP-pyrenyl-actin filaments than Mg-ADP-P_i_-pyrenyl-actin filaments and might contribute to the difference in pyrene fluorescence.

### Interactions of myosin and cofilin with pyrenyl-actin filaments and profilin with pyrenyl-actin monomers

**Fig. 6:**
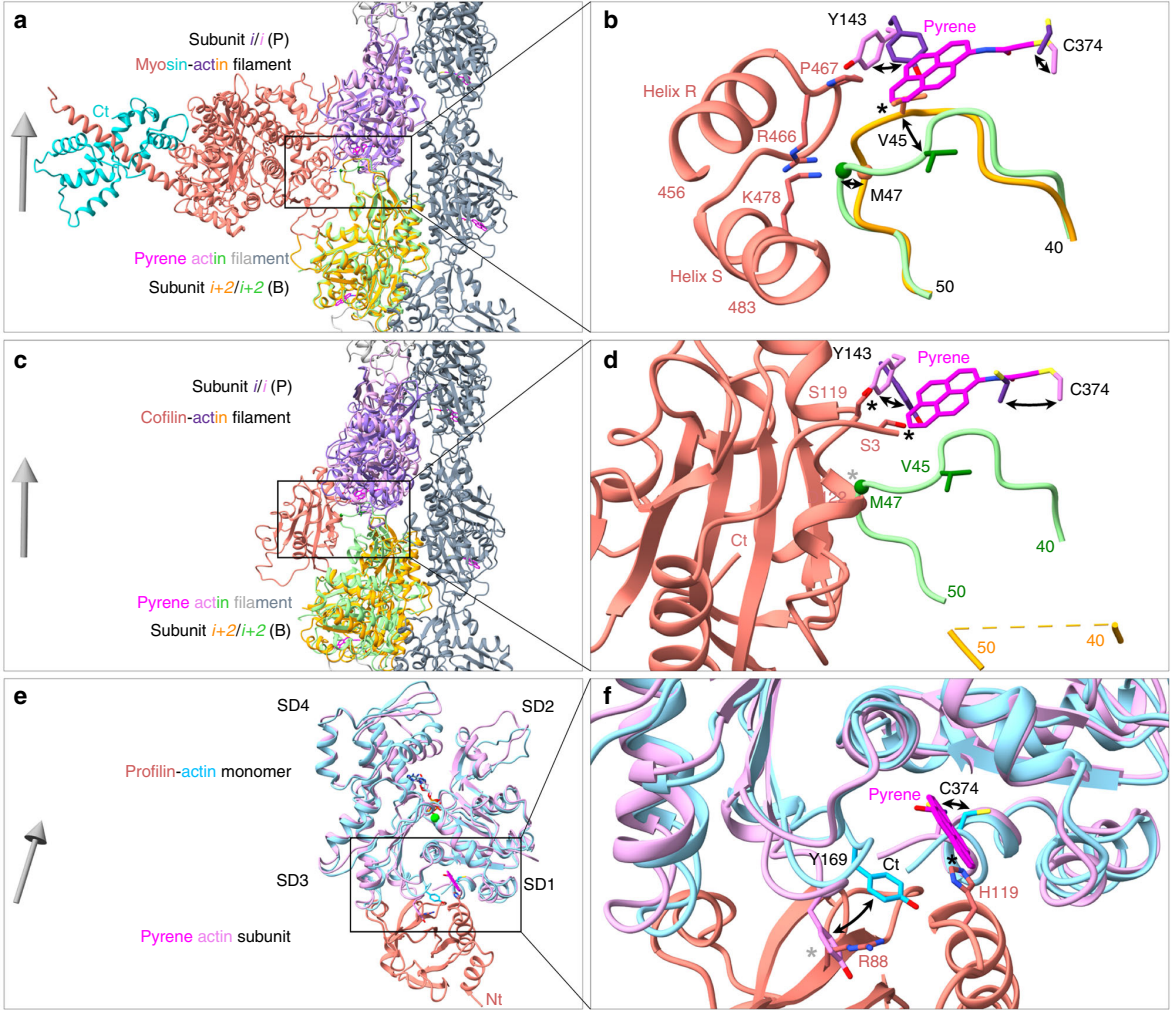
Ribbon diagrams showing the influence of pyrene labeling on the interactions of myosin and cofilin with actin filaments and of profilin with actin monomers. Gray arrows show the filament orientation with arrowheads pointing to the pointed end. Black curved arrows show conformational changes. Black stars label strong clashes; gray stars mark local conformational changes that cause minor or no clashes. **a**, **c**, **e** Overviews; **b**, **d**, **f** simplified close-up views. **a**, **b** Superimposition of an ADP-actin filament decorated with myosin-Ib (PDB ID: 6C1D) on our ADP-pyrenyl-actin filament. All the C_α_ atoms of subunit *i* in these two filaments were used for the superimposition. The presence of pyrene would cause two steric clashes with myosin: rotation of the side chain of Y143 of actin would clash with the cyclic side chain of P467 of the myosin heavy chain; and the backbone of actin M47 clashes with the side chains of myosin R466 and K478. Binding of myosin to pyrenyl-actin filament is likely to displace pyrene partially from its hydrophobic pocket. **c**, **d** Superimposition of a cofilin-decorated ADP-actin filament (PDB ID: 5YU8) on our ADP-pyrenyl-actin filament. The C_α_ atoms of residues in subdomain 3 of subunit *i* were used for the superimposition. The presence of pyrene would cause two steric clashes with cofilin: the side chain of actin Y143 clashes with the side chain of cofilin S119; and the pyrene group clashes with the side chain of cofilin S3. Binding of cofilin to ADP-pyrenyl-actin filament would squeeze part of the pyrene group out of the hydrophobic pocket and displace the D-loop of the neighboring subunit. **e**, **f** Superimposition of a profilin-actin monomer complex (PDB ID: 2BTF) on a subunit in our ADP-pyrenyl-actin filament using subdomains 1 and 3. The C_α_ atoms of residues in subdomains 1 and 3 are used for their superimposition. The side chain of profilin H119 would clash with the pyrene group. The side chain of actin Y169 could avoid the clash with the side chain of R88 in profilin by rearranging the conformation of the Y169 loop (WH2-binding loop). SD1-4: subdomains 1-4.

The original application of pyrenyl-actin filaments was to measure myosin binding^1^, because bound myosin reduces the fluorescence intensity by ~30%. The presence of pyrene also reduces the affinity of myosin for actin filaments by about half^9^. Superimposing the structure of a native ADP-actin filament decorated with myosin-Ib (PDB ID: 6C1D) onto our ADP-pyrenyl-actin filament showed that the presence of pyrene forces the side chain of actin Y143 into a position that slightly clashes with the side chain of myosin P467 and the backbone of actin M47 into a minor clash with the side chains of myosin R466 and K478 (Fig. 6b). Reciprocally, when myosin is bound to actin, the region of the actin D-loop around V45 would clash with the pyrene (Fig. 6b). Therefore, strong binding of myosin to actin filaments likely forces Y143 and the D-loop to displace the pyrene partially from its hydrophobic pocket into the solvent, accounting for the lower fluorescence.

Cofilin binding reduces the fluorescence of Mg-ADP-pyrenyl-actin filaments more than ten-fold without depolymerization^4,5^. Superimposing the structure of one subunit from a cofilin-decorated native ADP-actin filament (PDB ID: 5YU8) onto our ADP-pyrenyl-actin filament showed that the presence of pyrene would cause two strong steric clashes with cofilin: the side chain of actin Y143 clashes with the side chain of cofilin S119; and the pyrene group clashes with the side chain of cofilin S3 (Fig. 6d). To avoid this steric clash, cofilin must push Y143 toward the pyrene, which would displace pyrene from the hydrophobic pocket (Fig. 6d). This exposure of the pyrene group in the cofilin-decorated ADP-pyrenyl-actin filament would likely reduce its quantum yield. Furthermore, most of the D-loop residues (41–49) are disordered in cofilin-decorated ADP-actin filaments, eliminating the bottom of the hydrophobic pocket for pyrene.

Profilin binding enhances the fluorescence of pyrenyl-actin monomers^3^, but the pyrene label reduces the affinity of actin monomers for profilin by an order of magnitude^10^. Superimposing a crystal structure of profilin bound to native ATP-actin monomer (PDB ID: 2BTF) onto a subunit from our ADP-pyrenyl-actin filament showed the differences expected due to subunit flattening in the filament. The comparison also showed that the side chain of H119 in profilin would clash with the pyrene group (Fig. 6f), accounting for the lower affinity of profilin for pyrenyl-actin monomers. The side chain of actin Y169 could avoid the clash with the side chain of profilin R88 by rearranging the conformation of the Y169 loop (WH2-binding loop) (Fig. 6f). This increases the buried surface area of pyrene from 295 Å^2^ in the actin monomer to 356 Å^2^ in the profilin-actin monomer. This together with a more hydrophobic environment for the pyrene may account for the modestly higher fluorescence.

## Discussion

Mechanism of fluorescence enhancement by polymerization of pyrenyl-actin monomers: the change of environment from a solvent-exposed position to a hydrophobic pocket between the subunits in a filament explains why the fluorescence intensity of pyrenyl-actin increases ~20-fold upon polymerization. Pyrene fits almost perfectly into this hydrophobic pocket but displaces the side chain and backbone of V45. The side chain of M44 is the most important residue for inter-subunit interactions along the long-pitch helix. The presence of the pyrene group moves the backbone C_α_ atom of M44 toward the filament barbed end by 0.7 Å, but its side chain remains in place. These small changes do not alter the critical concentration or kinetics of spontaneous polymerization^1,2^.

On the other hand, labeling actin residue C374 with tetramethylrhodamine or Oregon green (Supplementary Fig. 5) alters the rates of Mg-ATP-actin monomers binding and dissociating at the ends of actin filaments with the changes depending on the fraction of labeled subunits^11,12^. Both of these fluorescent dyes are much larger than pyrene, so they cannot fit into the hydrophobic pocket between subunits along the long-pitch helix and are likely to clash with the adjacent subunit. The positive charge on tetramethylrhodamine may contribute to inhibiting elongation more strongly than Oregon green. Labeling lysine with a fluorescent dye in filaments of actin has smaller effects on polymerization^11,12^.

Prior to the introduction of pyrenyl-actin, actin labeled with Chloro-4-nitrobenzeno-2-oxa-1,3-diazole (NBD-Cl) was used to measure actin polymerization spectroscopically. NBD-Cl can modify the amine groups of lysines and arginines as well as reacting with the thiol group of cysteines. NBD conjugates of thiols are often unstable, so NBD-actin was prepared by first blocking C374 with N-ethylmaleimide (NEM) followed by labeling actin K373 with NBD-Cl^13^. Polymerization increases the fluorescence of NBD-actin (modified at C374 with NEM and at K373 with NBD-Cl) about 2.3-fold. In ADP-P_i_-pyrenyl-actin filaments, the primary amine group on the side chain of K373 is >10 Å from the hydrophobic pocket that encloses pyrene conjugated to C374 (Supplementary Fig. 6), so NBD conjugated to K373 is unlikely to occupy the pyrene binding pocket.

Mechanisms of fluorescence quenching by myosin and cofilin: binding of both myosin and cofilin to pyrenyl-actin filaments involves steric clashes that do not occur in native actin filaments (Fig. 6b, d). These clashes likely result in local conformational changes that expose the pyrene to solvent and explain why both proteins lower the fluorescence and why the presence of pyrene reduces the affinities of both proteins for actin filaments as explained in the results section.

Profilin binding enhances the fluorescence of pyrenyl-actin monomers. The likely explanation is reducing the exposure of pyrene to the solvent (Fig. 6f). Minor steric clashes between profilin and actin labeled with pyrene explain why the dye reduces the affinity ten-fold.

Effect of phosphate on the labeling and fluorescence of ADP-actin filaments: phosphate associated with ADP in the active site influences both the reaction of N-(1-pyrene) iodoacetamide with actin filaments and the fluorescence of pyrenyl-actin filaments. In our high-resolution EM structures of native actin filaments^7^, C374 is slightly more exposed in the ADP-actin filament than the ADP-P_i_-filament, explaining the different labeling rates. The explanation for the effect of phosphate on the fluorescence of ADP-pyrenyl-actin filaments is less clear, but may be due to the pyrene being trapped more rigidly in the hydrophobic pocket of filaments with phosphate. Lower map densities for the link between C374 and pyrene and around G48 and higher B-factors are evidence of greater mobility in Mg-ADP-pyrenyl-actin filaments.

Chemical and post-translational modifications of C374: actin C374 can be post-translationally modified by oxidation, glutathionylation, carbonylation, and nitrosylation^14^. These modifications on C374 usually decrease the polymerization rate, increase the critical concentration, weaken filament stability, and alter actin functions. These modifying chemical groups, especially large hydrophobic groups like glutathione, may occupy the space where pyrene binds.

## Methods

### Materials

We purchased N-(1-pyrene)iodoacetamide (sc-472510) from Santa Cruz Biotechnologies, ATP (A2383), ADP (A2754), hexokinase (H6380) from Sigma-Aldrich, and glucose (167454) from Thermo Fisher Scientific.

### Actin Purification

Chloroform-washed actin acetone powder was made from the flash-frozen chicken skeletal muscle^15^ purchased from a local Trader Joe’s grocery store. Actin was extracted from the powder with buffer G (2 mM Tris-HCl, pH 8.0, 0.1 mM CaCl_2_, 0.1 mM ATP, 0.5 mM DTT, 1 mM NaN_3_) for 30 min at 4 °C, and polymerized by adding KCl to 50 mM and MgCl_2_ to 2 mM. Actin filaments were pelleted by centrifugation at 140,000 × *g* for 2 h at 4 °C and depolymerized by dialysis against four changes of buffer G at 4 °C over 72 h. The sample was centrifuged at 140,000 × *g* for 2 h at 4 °C. The top 2/3 of depolymerized actin supernatant was purified by gel filtration on a Sephacryl S-300 column equilibrated with buffer G^16^. The concentration of native actin monomers was determined by absorbance at 290 nm using [actin, µM] = A_290_ × 38.5.

### Labeling ADP-actin filaments with N-(1-pyrene) iodoacetamide

The bottom 1/3 of depolymerized actin supernatant was polymerized by dialyzing for 10 h against three changes of PL buffer (100 mM KCl, 2 mM MgSO_4_, 25 mM Tris-HCl, pH 7.5, 0.3 mM ATP, 3 mM NaN_3_). During polymerization, bound ATP was hydrolyzed into ADP and phosphate (P_i_). Dissociated P_i_ and DTT (from buffer G) were removed during dialysis. The repolymerized actin was diluted to 1 mg/mL (23.8 µM) with PL buffer and incubated at 4 °C for 36 h with 0.15 mM N-(1-pyrene)iodoacetamide from a stock solution of 10 mM dissolved in dimethylformamide. Labeled actin filaments were collected by centrifugation, the pellet was homogenized in buffer G, and the mixture dialyzed against buffer G for 2 days at 4 °C. After clarification at 4 °C in a Beckman Ti70 rotor at 148,229 × *g* for 2 h, the top 80% of supernatant was gel filtered through a Superdex 200 column equilibrated with buffer G. The concentrations of pyrene and actin in the peak tail fractions were determined by measuring the absorbances at 344 nm (A_344_) and 290 nm (A_290_) and using these formulas: [pyrene, µM] = A_344_ × 45.5 µM/OD and [actin, µM] = (A_290_ – (A_344_ × 0.127)) × 38.5 µM/OD^1,2^. The ratio of pyrene/actin was close to 1.0. Both native and pyrenyl actin monomers were stored at 4 °C and used in less than a week.

Ca^2+^ bound to actin monomers was exchanged for Mg^2+^ by incubating with 0.1 volumes of 10× ME buffer (0.5 mM MgCl_2_, 2 mM EGTA, pH 7.5) at room temperature for 10 min. Mg-ATP-pyrenyl-actin was converted to Mg-ADP-pyrenyl-actin by incubating with 1 mM glucose and 5 units/mL hexokinase at room temperature for 30 min, followed by adding 1 mM ADP, pH 7.0 and polymerizing the actin for 2 h at 25 °C by adding 0.1 volumes of 10× KMEI buffer (1 M KCl, 10 mM MgCl_2_, 10 mM EGTA, 100 mM imidazole, pH 7.0, 5 mM DTT) to make ADP-actin filaments or first adding potassium phosphate (pH 7.0) to 30 mM and then 0.1 volumes of 10× KMEI buffer to make ADP-P_i_-actin filaments. Pyrenyl actin filaments were stored at 4 °C for 10 h before vitrification for EM analysis.

### Fluorescence assay to measure pyrene labeling and phosphate binding

Mg-ADP-actin filaments (94 µL; 10.7 µM) in PLD buffer (100 mM KCl, 2 mM MgCl_2_, 10 mM imidazole, pH 7.0, 0.3 mM ADP, 3 mM NaN_3_) were preincubated by adding 6 µL of either H_2_O, 250 mM potassium phosphate pH 7.0, or 250 mM K_2_SO_4_ pH 7.0 for 10 min before adding 50 µL of 150 µM N-(1-pyrene)iodoacetamide diluted from the above-mentioned stock solution with PLD buffer. The time course of the fluorescence change was measured in a Costar black polystyrene 96-well plate (3694; Corning Incorporated, Corning, NY) using a SpectraMAX plate reader (Genimi EM; Molecular Devices, San Jose, CA) with excitation at 365 nm and emission at 407 nm. The plate reader was controlled by SoftMax.

To measure the binding of phosphate to Mg-ADP-pyrenyl-actin filaments, 120 µL of 5 µM Mg-ADP-pyrenyl-actin filaments, and 30 µL of phosphate or sulfate at various concentrations were incubated for ~1 h before measuring the fluorescence with the same plate reader mentioned above. Mean values and standard deviations were calculated from five readings on the same samples. The fluorescence graphs (Fig. 4) were plotted with OriginLab.

### Sample Freezing and Image Collectio**n**

Holy carbon Quantifoil 2/1 300-mesh gold grids (Electron Microscopy Sciences, Hatfield, PA) were glow-discharged for 30 s in a Bal-Tec SCD 005 sputter coater (Leica Biosystem Inc.) in air (pressure: 0.05 mBar) at 25 mA. We applied 3 µL of 18 µM of pyrenyl actin filaments to the carbon side of the grids in a Vitrobot Mark IV (FEI company, Hillsboro, OR) at 10 °C and 100% humidity. After waiting for 40 s, the extra solution was blotted off using Vitrobot standard filter paper (grade: 595; Ted Pella, Redding, CA) for 2.5 s at blot force −15. Grids were frozen by plunging into liquid ethane cooled to about −180 °C and screened on a Talos electron microscope operated at 120 kV and equipped with a Ceta 16 M camera (FEI company, Hillsboro, OR). The two datasets were collected in a consecutive session on a Titan Krios microscope equipped with an XFEG at 300 kV, a nanoprobe, and a Gatan image filter (slit width: 20 eV). Movies were recorded at a series of defocus values between −1.0 µm and −2.5 µm on a K2 summit camera in super-resolution mode, using the beam image shift strategy (4 movies/hole) designed in the third-party program SerialEM^17^. Each movie had 55 frames and each frame time was 0.20 s. The dose rate on camera level was 5.6 counts/pixel/s, and the physical pixel size was 1.05 Å. After magnification distortion correction, the pixels size became 1.045 Å.

We collected 1560 low-dose movies of Mg-ADP-P_i_-actin filaments and 1980 low-dose movies of Mg-ADP-pyrenyl-actin filaments. The ADP-actin filament samples (critical concentration: 0.8 µM) had more actin monomers in the background than the ADP-P_i_-actin filament sample (critical concentration: 0.1 µM) (Supplementary Fig. 1a, b).

### EM Map Reconstruction

Movies were dose-averaged, magnification-corrected, motion-removed, and summed with MotionCor2^18^ using 9 × 9 patches. Parameters in the contrast transfer function (CTF) were estimated with Gctf^19^ using summed but unweighted micrographs. Filaments were first autopicked in RELION^20^ with a curve factor of 0.95. The coordinates were exported in box format for manual adjustment with sxhelixboxer.py in SPARX^21^. The manually-adjusted filament coordinates were read back into RELION for further analysis. Before helical reconstruction, the filaments were windowed into overlapping square segments by translating the box (328 × 328 pixels) along the central axis of each filament by one subunit (26 pixels). Each segment contained ~12 subunits. Our map of native ADP-P_i_-actin filaments (EMD-7937) was low-pass filtered at 10 Å before being used as the initial map. Particle local CTFs were used in the reconstructions. A soft-edged 3D mask with a radius of 45% of the box size was created for postprocessing. The B-factors for map sharpening were determined by RELION. A cutoff of 0.143 was used for resolution estimation using the Fourier shell correlation method. At this point, the map of Mg-ADP-P_i_-pyrenyl-actin filaments was refined to 3.3 Å, and that of Mg-ADP-pyrenyl-actin filaments was 3.5 Å.

Because the filaments are continuous, the particle local CTFs should change continuously along each filament. However, the particle local CTFs varied along most of the filaments owing to the low signal-to-noise ratio in the images, the nature of electron micrographs. Therefore, we smoothed the CTF of each particle along the same filament locally over five neighboring particles. The highest and lowest CTFs were removed and the remaining three were fitted using linear regression. After this smoothing step, most of the particle local CTFs changed continuously along each filament.

We calculated the rise and twist for the consistently aligned particles (>99.8%) using our own program, which is available on the source code management website GitHub https://github.com/stevenzchou/SoFiPa, and then calculated their mean and standard deviation. After removing the rise and twist outliers (>2× SD), we reconstructed a new map using particles in a certain confidence interval (95%). The best reconstructions were obtained after a trade-off between particle number and assembly homogeneity. This process improved the resolutions of the maps to 3.0 Å for Mg-ADP-P_i_-pyrenyl-actin filaments and to 3.2 Å for Mg-ADP-pyrenyl-actin filaments (Supplementary Fig. 2; Supplementary Table 1). The Fourier shell correlation curves were plotted with GnuPlot. Layer-line images were generated with SPARX^21^. Local resolutions were estimated with ResMap^22^. All the calculations were run on the Farnam computer cluster maintained by Yale High Performance Computing team.

### Model building, refinement, and visualization

Our EM structure of native ADP-P_i_-actin filaments (PDB ID: 6DJN) was used as the initial model. The chemical restraints for the pyrene moiety in cif format were generated with eLBOW tool in Phenix^23,24^. The atomic coordinates for the pyrene moiety were named according to the crystal structure of N-(1-pyrene)acetamide-labeled P450 (PDB ID: 4KKY). The coordinates of actin and pyrene were joined together manually in a text editor, and the pyrene group was moved close to C374 of each actin subunit in Coot^25^ to get the starting model of pyrenyl-actin filaments. Further restraints for linking the pyrene moiety to actin in edits format were added manually. The coordinates were refined against the EM maps of pyrenyl-actin filaments in real space with Phenix^26^ and checked with Coot. Figures of maps and models were generated with ChimeraX^27^. The electrostatic potentials were calculated with DelPhi^28^, and mapped onto the molecular surfaces in ChimeraX using an offset of 0 Å. Root mean square deviations and buried surface areas were calculated with Chimera^29^. PyMOL^30^ was used to calculate the solvent-accessible surface areas and to map the B-factors onto the cartoon diagram. The structural formulas of small compounds (Supplementary Figs. 5 and 6) were generated with ChemDraw.

### Reporting summary

Further information on research design is available in the Nature Research Reporting Summary linked to this article.

## Supplementary information

Supplementary Information

Reporting Summary

## Data Availability

Data supporting the findings of this manuscript are available from the corresponding authors upon reasonable request. A reporting summary for this article is available as a Supplementary Information file. Source data are provided with this paper. The cryo-EM density maps from this study have been deposited in the Electron Microscopy Data Bank (EMDB) under accession numbers EMD-22639 for ADP-P_i_-pyrenyl-actin filaments, EMD-22638 for ADP-pyrenyl-actin filaments. The coordinates of the atomic models have been deposited in the Protein Data Bank (PDB) under accession numbers PDB 7K21 for ADP-P_i_-pyrenyl-actin filaments, PDB 7K20 for ADP-pyrenyl-actin filaments.

## Code availability

The computer code of SoFiPa v1.0 (written in C++) is openly available from GitHub at https://github.com/stevenzchou/SoFiPa.

## References

[1] Kouyama T, Mihashi K (1981). Fluorimetry study of N-(1-pyrenyl)iodoacetamide-labelled F-actin. Local structural change of actin protomer both on polymerization and on binding of heavy meromyosin. Eur. J. Biochem..

[2] Cooper JA, Walker SB, Pollard TD (1983). Pyrene actin: documentation of the validity of a sensitive assay for actin polymerization. J. Muscle Res. Cell Motil..

[3] Lee S, Li M, Pollard TD (1988). Evaluation of the binding of Acanthamoeba profilin to pyrene-labeled actin by fluorescence enhancement. Anal. Biochem..

[4] Nishida E, Maekawa S, Sakai H (1984). Cofilin, a protein in porcine brain that binds to actin filaments and inhibits their interactions with myosin and tropomyosin. Biochem.

[5] Carlier MF (1997). Actin depolymerizing factor (ADF/cofilin) enhances the rate of filament turnover: implication in actin-based motility. J. Cell Biol..

[6] Merino F (2018). Structural transitions of F-actin upon ATP hydrolysis at near-atomic resolution revealed by cryo-EM. Nat. Struct. Mol. Biol..

[7] Chou SZ, Pollard TD (2019). Mechanism of actin polymerization revealed by cryo-EM structures of actin filaments with three different bound nucleotides. Proc. Nat. Acad. Sci. USA.

[8] Carlier MF, Pantaloni D (1986). Direct evidence for ADP-Pi-F-actin as the major intermediate in ATP-actin polymerization. Rate of dissociation of Pi from actin filaments. Biochemistry.

[9] Taylor EW (1991). Kinetic studies on the association and dissociation of myosin subfragment 1 and actin. J. Biol. Chem..

[10] Vinson VK, De La Cruz EM, Higgs HN, Pollard TD (1998). Interactions of Acanthamoeba profilin with actin and nucleotides bound to actin. Biochemistry.

[11] Amann KJ, Pollard TD (2001). The Arp2/3 complex nucleates actin filament branches from the sides of pre-existing filaments. Nat. Cell Biol..

[12] Fujiwara I, Zweifel ME, Courtemanche N, Pollard TD (2018). Latrunculin A accelerates actin filament depolymerization in addition to sequestering actin monomers. Curr. Biol..

[13] Detmers P, Weber A, Elzinga M, Stephens RE (1981). 7-Chloro-4-nitrobenzeno-2-oxa-1,3-diazole actin as a probe for actin polymerization. J. Biol. Chem..

[14] Terman JR, Kashina A (2013). Post-translational modification and regulation of actin. Curr. Opin. Cell Biol..

[15] Feuer G, Straub FB (1948). Studies on the composition and polymerization of actin. Hung. Acta Physiol..

[16] MacLean-Fletcher S, Pollard TD (1980). Identification of a factor in conventional muscle actin preparations which inhibits actin filament self-association. Biochem. Biophys. Res. Commun..

[17] Mastronarde DN (2005). Automated electron microscope tomography using robust prediction of specimen movements. J. Struct. Biol..

[18] Zheng SQ (2017). MotionCor2: anisotropic correction of beam-induced motion for improved cryo-electron microscopy. Nat. Methods.

[19] Zhang K (2016). Gctf: Real-time CTF determination and correction. J. Struct. Biol..

[20] He S, Scheres SHW (2017). Helical reconstruction in RELION. J. Struct. Biol..

[21] Hohn M (2007). SPARX, a new environment for Cryo-EM image processing. J. Struct. Biol..

[22] Kucukelbir A, Sigworth FJ, Tagare HD (2014). Quantifying the local resolution of cryo-EM density maps. Nat. Methods.

[23] Adams PD (2010). PHENIX: a comprehensive Python-based system for macromolecular structure solution. Acta Crystallogr. D. Biol. Crystallogr..

[24] Moriarty NW, Grosse-Kunstleve RW, Adams PD (2009). electronic Ligand Builder and Optimization Workbench (eLBOW): a tool for ligand coordinate and restraint generation. Acta Crystallogr. D. Biol. Crystallogr..

[25] Emsley P, Lohkamp B, Scott WG, Cowtan K (2010). Features and development of coot. Acta Crystallgr. D Biol. Crystallogr.

[26] Afonine PV (2018). Real-space refinement in PHENIX for cryo-EM and crystallography. Acta Crystallogr D. Struct. Biol..

[27] Goddard TD (2018). UCSF ChimeraX: Meeting modern challenges in visualization and analysis. Protein Sci..

[28] Rocchia W (2002). Rapid grid-based construction of the molecular surface and the use of induced surface charge to calculate reaction field energies: applications to the molecular systems and geometric objects. J. Comput. Chem..

[29] Pettersen EF (2004). UCSF Chimera–a visualization system for exploratory research and analysis. J. Comput. Chem..

[30] Schrodinger, L. The PyMOL Molecular Graphics System, Version 1.8 (2015).

