# Supplementary material for: Cryo-electron microscopy structures of pyrene-labeled ADP-Pi- and ADP-actin filaments
Source: Nat Commun. 2020 Nov 19;11:5897. doi: 10.1038/s41467-020-19762-1 (PMC7677365; doi:10.1038/s41467-020-19762-1)
Supplement: Supplementary file 1 — Supplementary Information [file 41467_2020_19762_MOESM1_ESM.pdf]

Supplementary Information

Cryo-electron microscopy structures of pyrene-labeled ADP-P_i- and ADP-actin filaments

Steven Z. Chou[1] and Thomas D. Pollard[1,2,3,*]

[1]Department of Molecular Cellular and Developmental Biology
[2]Department of Molecular Biophysics and Biochemistry
[3]Department of Cell Biology
Yale University, PO Box 208103, New Haven, CT 06520-8103 USA
*Correspondence: thomas.pollard@yale.edu

Key words: actin, cryo-electron microscopy, fluorescence, pyrene

Supplementary Table 1. Statistics for data collection, map reconstruction and model refinement.

	Mg-ADP-P_i-pyrenyl actin filament (EMD-22639, PDB 7K21)	Mg-ADP-pyrenyl actin filament (EMD-22638, PDB 7K20)
**Data collection and map reconstruction**		
Magnification	47619	47619
Voltage (kV)	300	300
Electron exposure (e^-/Å^2)	55.87	55.87
Defocus range (μm) (set/measured)	[-2.5, -1.5]/[-3.2, -1.3]	[-2.5, -1.5]/[-3.5, -1.3]
Pixel size (Å)	1.045	1.045
Rise (Å)/twist (°)/Symmetry	27.37/-166.57/C1	27.39/-166.58/C1
Initial particle images (no.)	462669	268618
Final particle images (no.)	411301	240254
Map resolution (Å)	3.0	3.2
FSC threshold	0.143	0.143
Map resolution range (Å)	2.7-3.3	2.7-3.6
Map sharpening B factor (Å^2)	-100.43	-114.39
**Model Refinement**		
Initial model used (PDB ID)	6DJN	6DJN
Model resolution (Å)	3.07	3.27
FSC threshold	0.5	0.5
Model composition		
Heavy atoms	11848	11828
Protein residues	1488	1488
Ligands (ADP/P_i/Mg^{2+}/pyrene)	4/4/4/4	4/0/4/4
B factors (Å^2)		
Protein	75.51	74.31
Ligands	70.84	70.21
R.m.s. deviations		
Bond lengths (Å)	0.007	0.013
Bond angles (°)	0.788	0.953
Geometry validation		
MolProbity score	1.38	1.45
Clashscore	5.12	3.46
Poor rotamers (%)	0.00	0.00
Ramachandran plot		
Favored (%)	97.48	95.50
Allowed (%)	2.52	4.50
Disallowed (%)	0.00	0.00

Supplementary Table 2. Comparison of filament rise and twist in the final models and each filament particle.

Sample	Rise in final model	Rise in each particle
	Twist in final model	Twist in each particle
ADP-P_i-pyrenyl-actin filaments	27.380 ± 0.001	27.383 ± 0.687
	-166.579 ± 0.001	-166.641 ± 0.819
ADP-P_i-actin filaments	27.329 ± 0.002	27.377 ± 0.389
	-166.528 ± 0.003	-166.637 ± 0.619
ADP-pyrenyl-actin filaments	27.396 ± 0.001	27.234 ± 0.822
	-166.596 ± 0.001	-166.652 ± 0.836
ADP-actin filaments	27.518 ± 0.002	27.388 ± 0.479
	-166.625 ± 0.001	-166.607 ± 0.705

Note: Values are in the format of mean ± standard deviation. The data for pyrenyl-actin filaments are from this study, and those for native actin filaments are from our previous study[1].

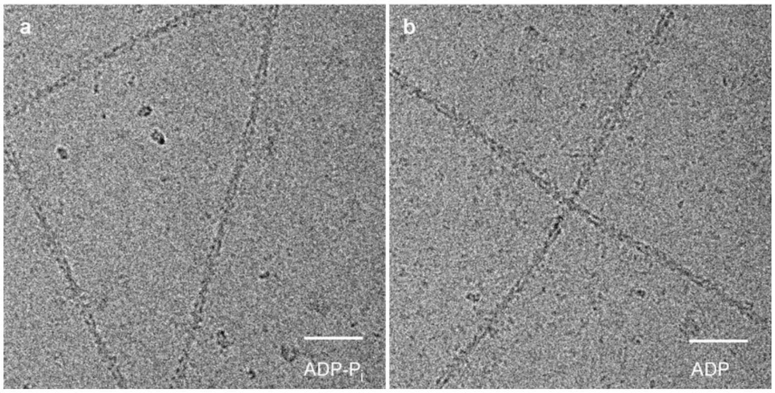

Supplementary Fig. 1 Micrographs of vitrified pyrenyl-actin filaments. a A representative area of ADP-P_i-pyrenyl-actin micrograph. **b** A representative area of ADP-pyrenyl-actin micrograph. ADP-pyrenyl-actin has more unpolymerized monomers. Scale bars: 50 nm. The appearance of electron micrographs is modulated by the defocus value. As long as the defocus was roughly between -2 µm and -3 µm, we were able to get micrographs like these two in most grid holes.

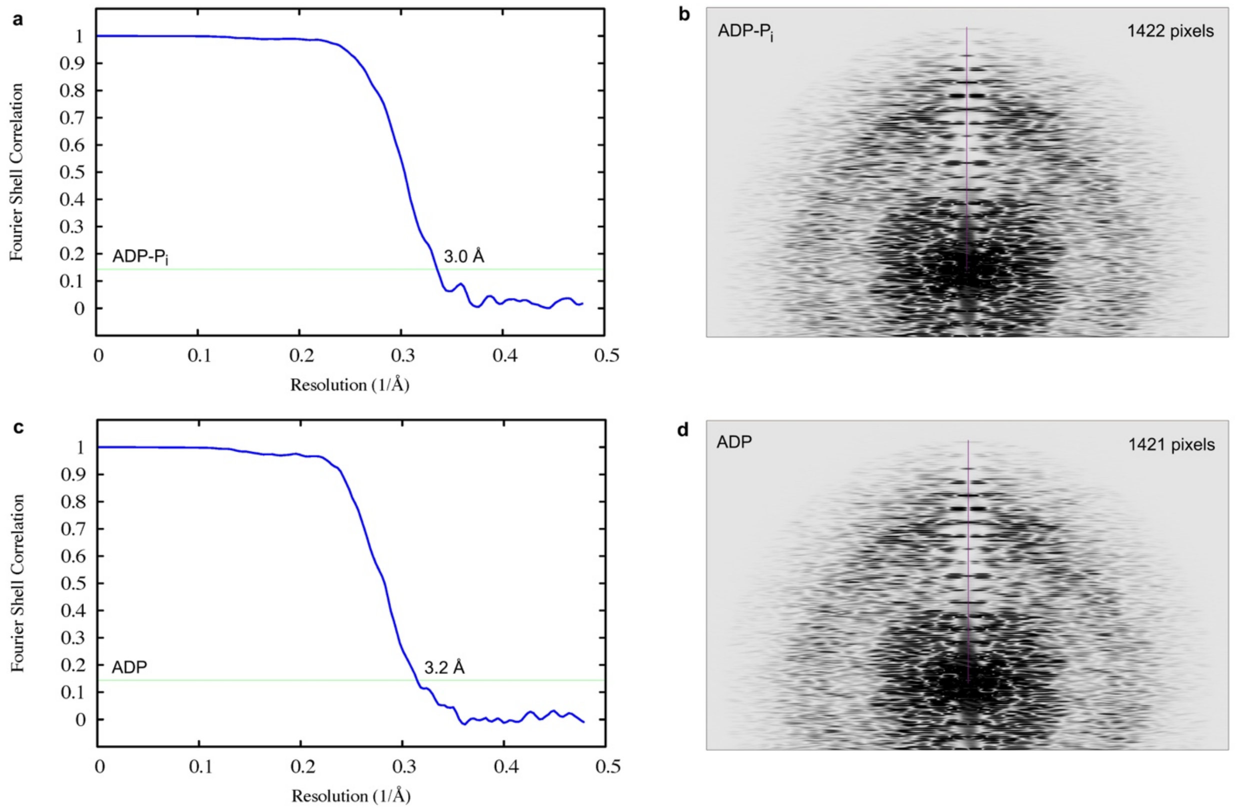

Supplementary Fig. 2 Global map resolution estimation. a, **c** Resolution of (**a**) ADP-P_i- and (**c**) ADP-pyrenyl-actin maps estimated using Fourier shell correlation (FSC, blue curves) with 0.143 criterion (blue horizontal lines). The estimated resolution is 3.0 Å for ADP-P_i-pyrenyl-actin filaments and 3.2 Å for ADP-pyrenyl-actin filaments. **b**, **d** Resolution of (**b**) ADP-P_i- and (**d**) ADP-pyrenyl-actin maps estimated using layer-line images calculated from map projections. The purple line is from the center of the layer-line image to the highest visible layer line. The size of layer-line images is 4096×4096 pixels, and the pixel size is 1.045 Å. Using the formula, resolution = (pixel size)×(layer-line image size)/(layer-line height), the estimated resolution is 3.0 Å for both ADP-P_i- and ADP-pyrenyl-actin filaments, in good agreement with the resolutions estimated by FSC.

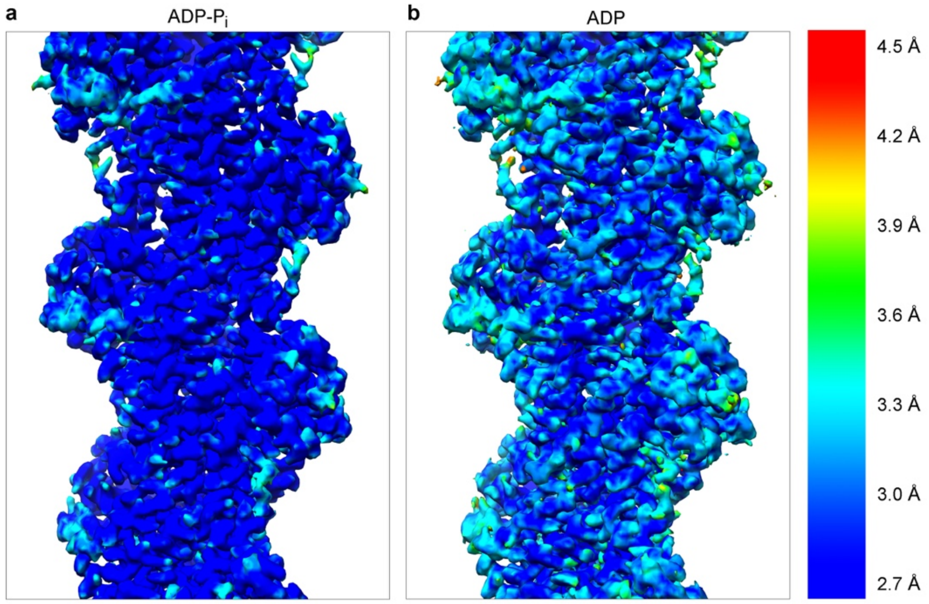

Supplementary Fig. 3 Local resolution estimation with ResMap. a Local resolution of ADP-P_i-pyrenyl-actin filament. Resolution mostly falls in the range of 2.7 Å and 3.3 Å. **b** Local resolution of ADP-pyrenyl-actin filament. Resolution mostly falls in the range of 2.7 Å and 3.6 Å.

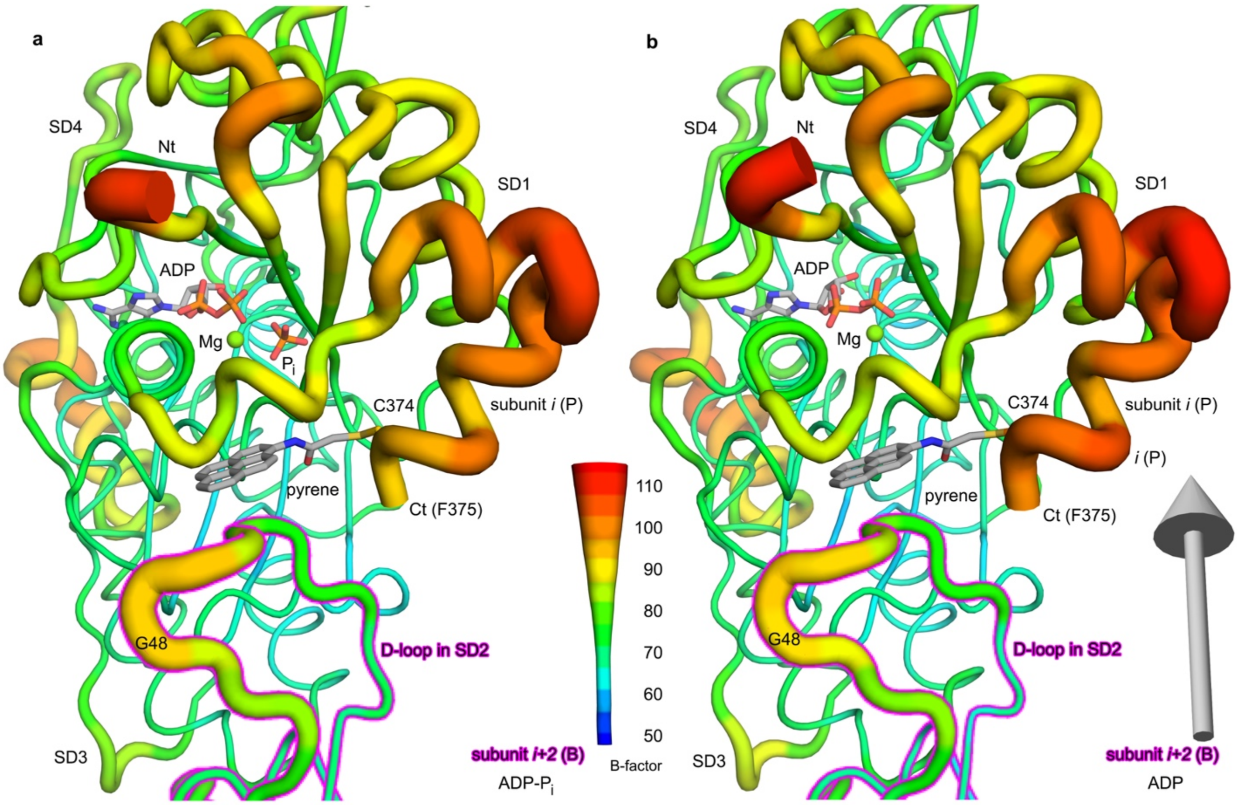

Supplementary Fig. 4 C-termini of actin subunits are more flexible in ADP-pyrenyl-actin filaments than the ADP-P_i-pyrenyl-actin filaments. The backbone is rendered as a tube with a diameter proportional to the B-factor of each residue and a color (blue, cyan, green, yellow, orange, and red) based on the B-factor values according to the color scale. Subunit P and the D-loop of subunit B are displayed with the subunit B silhouetted in magenta. High B-factors indicate large motion amplitude. **a** ADP-P_i-pyrenyl-actin filament. **b** ADP-pyrenyl-actin filament. ADP, phosphate (P_i), pyrene and the side chain of C374 are shown as sticks, and cation (Mg) as a green ball. SD1-4: subdomains 1-4.

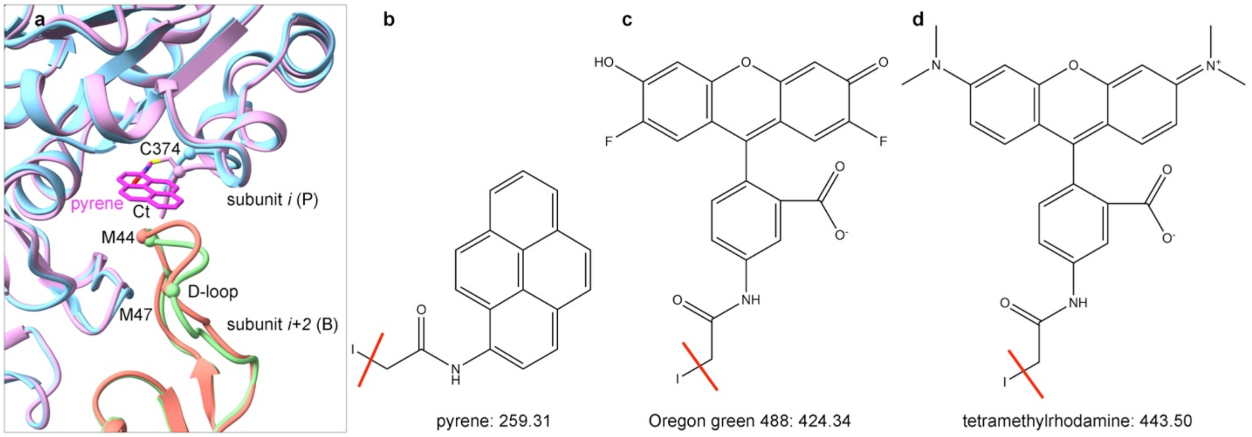

Supplementary Fig. 5 Pyrene binding site and structures of the commonly used fluorophores for labeling C374 of actin. a Conjugation of pyrene to C374 displaces residues between M44 and M47 in the D-loop. The two subunits (B and P) along the long-pitch helix in Mg-ADP-P_i-actin filament are colored in sky blue and salmon, and subunits in Mg-ADP-P_i-pyrenyl-actin filament are colored plum and light green. The pyrene group is colored in magenta. The C_α atoms of C374, M44 and M47 are shown as balls. **b** N-(1-pyrene)iodoacetamide (no charge). **c** Oregon green 488 iodoacetamide (a negative charge). (D) Tetramethylrhodamine iodoacetamide (a negative charge and a positive charge). During the conjugation reaction, iodine (on the left side of the red line) is removed. The molecular weight after the reaction is indicated below each structure.

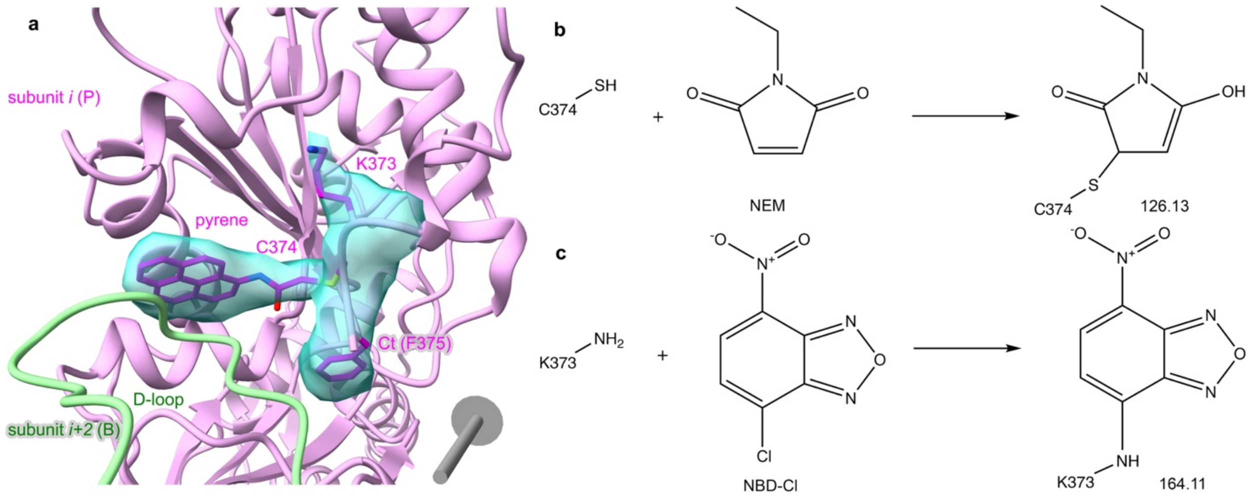

Supplementary Fig. 6 Location of the NBD-labeling site, K373, compared with the location of pyrene on C374 in the hydrophobic pocket between subunits. a Ribbon diagram of the ADP-P_i-pyrenyl-actin filament with EM densities as turquoise surface for pyrene and the C-terminal residues K373, C374 and F375. Subunit P is colored in plum, and subunit B in light green. Grey arrow shows the filament orientation with arrow head pointing to the pointed end. **b** The procedure to label actin filaments with 4-Chloro-7-nitrobenzo-2-oxa-1,3-diazole (NBD-Cl) involves blocking C374 with N-ethylmaleimide (NEM) before (**c**) labeling K373 with NBD-Cl[2]. The molecular weight after the reaction is indicated below each structure.

Supplementary References

1. Chou, S.Z. & Pollard, T.D. Mechanism of actin polymerization revealed by cryo-EM structures of actin filaments with three different bound nucleotides. *Proc Natl Acad Sci U S A* **116**, 4265-4274 (2019).
2. Detmers, P., Weber, A., Elzinga, M. & Stephens, R.E. 7-Chloro-4-nitrobenzeno-2-oxa-1,3-diazole actin as a probe for actin polymerization. *J Biol Chem* **256**, 99-105 (1981).
