# Supplementary material for: Cryo-electron microscopy structures of pyrene-labeled ADP-Pi- and ADP-actin filaments
Source: Nat Commun. 2020 Nov 19;11:5897. doi: 10.1038/s41467-020-19762-1 (PMC7677365; doi:10.1038/s41467-020-19762-1)
Supplement: Supplementary file 2 — Reporting Summary [file 41467_2020_19762_MOESM2_ESM.pdf]

nature research

Corresponding author(s): Thomas D. Pollard

Last updated by author(s): Sep 7, 2020

Reporting Summary

Nature Research wishes to improve the reproducibility of the work that we publish. This form provides structure for consistency and transparency in reporting. For further information on Nature Research policies, see our Editorial Policies and the Editorial Policy Checklist.

Statistics

For all statistical analyses, confirm that the following items are present in the figure legend, table legend, main text, or Methods section.

n/a	Confirmed	
☐	☒	The exact sample size (*n*) for each experimental group/condition, given as a discrete number and unit of measurement
☐	☒	A statement on whether measurements were taken from distinct samples or whether the same sample was measured repeatedly
☒	☐	The statistical test(s) used AND whether they are one- or two-sided<br>*Only common tests should be described solely by name; describe more complex techniques in the Methods section.*
☒	☐	A description of all covariates tested
☒	☐	A description of any assumptions or corrections, such as tests of normality and adjustment for multiple comparisons
☐	☒	A full description of the statistical parameters including central tendency (e.g. means) or other basic estimates (e.g. regression coefficient) AND variation (e.g. standard deviation) or associated estimates of uncertainty (e.g. confidence intervals)
☒	☐	For null hypothesis testing, the test statistic (e.g. *F*, *t*, *r*) with confidence intervals, effect sizes, degrees of freedom and *P* value noted<br>*Give P values as exact values whenever suitable.*
☒	☐	For Bayesian analysis, information on the choice of priors and Markov chain Monte Carlo settings
☒	☐	For hierarchical and complex designs, identification of the appropriate level for tests and full reporting of outcomes
☒	☐	Estimates of effect sizes (e.g. Cohen's *d*, Pearson's *r*), indicating how they were calculated

Our web collection on statistics for biologists contains articles on many of the points above.

Software and code

Policy information about availability of computer code

Data collection	SerialEM v3.7; SoftMax v7.0
Data analysis	MotionCor2 v1.2.1 (motion correction); Gctf v1.18 (CTF estimation); RELION v3.0.8 (map reconstruction); SPARX (particle picking); ResMap v1.95 (local resolution estimation); Phenix v1.12 (model refinement and validation); Coot v1.3.0 (model building); ChimeraX v1.0 (structure presentation); Chimera 1.10.1 (structure comparison); PyMOL v2.1.0 (SASA calculation); DelPhi v5.1 (electrostatic potential calculation); GnuPlot v4.6 (graph plotting); OriginLab (graph plotting;); ChemDraw v19.1 (presentation of small molecules)

For manuscripts utilizing custom algorithms or software that are central to the research but not yet described in published literature, software must be made available to editors and reviewers. We strongly encourage code deposition in a community repository (e.g. GitHub). See the Nature Research guidelines for submitting code & software for further information.

Data

Policy information about availability of data

All manuscripts must include a data availability statement. This statement should provide the following information, where applicable:
- Accession codes, unique identifiers, or web links for publicly available datasets
- A list of figures that have associated raw data
- A description of any restrictions on data availability

The cryo-EM density maps have been deposited in the Electron Microscopy Data Bank under accession numbers EMD-22639 for ADP-Pi-pyrenyl-actin filaments, EMD-22638 for ADP-pyrenyl-actin filaments. The coordinates of the atomic models have been deposited in the Protein Data Bank under accession numbers PDB 7K21 for ADP-Pi-pyrenyl-actin filaments, PDB 7K20 for ADP-pyrenyl-actin filaments.

April 2020

Field-specific reporting

Please select the one below that is the best fit for your research. If you are not sure, read the appropriate sections before making your selection.

☒ Life sciences ☐ Behavioural & social sciences ☐ Ecological, evolutionary & environmental sciences

For a reference copy of the document with all sections, see nature.com/documents/nr-reporting-summary-flat.pdf

Life sciences study design

All studies must disclose on these points even when the disclosure is negative.

Sample size	not relevant to structural experiments
Data exclusions	not relevant to structural experiments
Replication	We did not replicate structural experiments, which is standard practice.
Randomization	not relevant to structural experiments
Blinding	not relevant to structural experiments

Reporting for specific materials, systems and methods

We require information from authors about some types of materials, experimental systems and methods used in many studies. Here, indicate whether each material, system or method listed is relevant to your study. If you are not sure if a list item applies to your research, read the appropriate section before selecting a response.

Materials & experimental systems

n/a	Involved in the study	
☒	☐	Antibodies
☒	☐	Eukaryotic cell lines
☒	☐	Palaeontology and archaeology
☐	☒	Animals and other organisms
☒	☐	Human research participants
☒	☐	Clinical data
☒	☐	Dual use research of concern

Methods

n/a	Involved in the study	
☒	☐	ChIP-seq
☒	☐	Flow cytometry
☒	☐	MRI-based neuroimaging

Animals and other organisms

Policy information about studies involving animals; ARRIVE guidelines recommended for reporting animal research

Laboratory animals	N/A
Wild animals	We bought flash-frozen chicken muscle from a local Trader Joe's grocery store.
Field-collected samples	N/A
Ethics oversight	N/A

Note that full information on the approval of the study protocol must also be provided in the manuscript.
